# Association of Right Ventricular Dysfunction with Risk of Neurodevelopmental Impairment in Infants with Pulmonary Hypertension

**DOI:** 10.3390/children11091121

**Published:** 2024-09-13

**Authors:** Rossana Romero Orozco, Tazuddin A. Mohammed, Kerri Carter, Shaaron Brown, Stephen Miller, Roy T. Sabo, Meredith Campbell Joseph, Uyen Truong, Megha Nair, Victoria Anderson, Jie Xu, Judith A. Voynow, Karen D. Hendricks-Muñoz

**Affiliations:** 1 Department of Pediatrics, Children’s Hospital of Richmond at VCU, Virginia Commonwealth University, Richmond, VA 23298, USAkerri.carter@vcuhealth.org (K.C.); karen.hendircks-munoz@vcuhealth.org (K.D.H.-M.); 2 Virginia Commonwealth University School of Medicine, Richmond, VA 23298, USA; 3 Department of Physical Therapy, Children’s Hospital of Richmond at VCU, Virginia Commonwealth University, Richmond, VA 23298, USA; shaaron.brown@vcuhealth.org; 4 Department of Biostatistics, School of Public Health, Virginia Commonwealth University, Richmond, VA 23219, USA; 5 Department of Pediatrics, UF Health Shands Children’s Hospital, University of Florida, Gainesville, FL 32610, USA; 6 Department of Pediatrics, Children’s National Hospital, Washington, DC 20010, USA; 7 Sidney Kimmel Medical College, Thomas Jefferson University, Philadelphia, PA 19107, USA

**Keywords:** right ventricle (RV), pulmonary hypertension (PH), pulmonary vascular resistance (PVR), neurodevelopmental impairment (NI)

## Abstract

(1) Background: Pulmonary hypertension (PH) increases pulmonary vascular resistance and right ventricular (RV) afterload. Assessment of RV systolic function in PH using RV fractional area change (RV FAC) as a marker directly correlates with mortality and the need for extracorporeal membrane oxygenation (ECMO). However, few studies have assessed neurodevelopmental outcomes. We hypothesize that cardiac RV systolic dysfunction with lower RV FAC is associated with worse neurodevelopmental impairment (NI). (2) Methods: Retrospective study of 42 subjects with PH to evaluate neurodevelopmental outcomes in the first two years of life based on (i) subjective assessment of RV systolic function and (ii) RV FAC, a specific echocardiographic marker for RV function. (3) Results: Subjects from the initial study cohort (*n* = 135) with PH who had long-term follow-up were divided into RV dysfunction (study, *n* = 20) and non-RV dysfunction (control, *n* = 22) groups. RV FAC in the study vs. control group (0.18 vs. 0.25) was lower (*p* = 0.00017). There was no statistically significant difference in NI either with RV dysfunction or lower RV FAC. Although not significant, RV dysfunction was associated with longer mean duration of mechanical ventilation, time on ECMO, and length of stay. In the initial cohort (135), mortality was 16.3% and the percentage of NI was 62%. (4) Conclusions: Neonatal pulmonary hypertension is associated with a high degree of neurodevelopment impairment. Early RV systolic dysfunction, as identified by RV FAC, was not an optimal predictive biomarker for infants with PH and neurodevelopmental impairment.

## 1. Introduction

Pulmonary hypertension (PH) during the neonatal period results from the failure of transition from fetal to postnatal circulation, leading to a sustained elevation of pulmonary vascular resistance, causing an increase in right ventricle (RV) load that leads to RV failure and death [1]. The incidence of neonatal pulmonary hypertension is 2 per 1000 live births (0.4–6.8 per 1000 live births) in the United States and is ten times higher in developing countries [2]. The mortality in moderate and severe neonatal PH ranges from 4% to 33%, and surviving infants have an increased risk of long-term morbidities, with 25–46% having neurodevelopmental impairment (NI) by two years of age [3,4]. Etiologies of pulmonary hypertension include conditions that lead to abnormal pulmonary vasoreactivity (respiratory distress syndrome of the newborn, meconium aspiration syndrome, sepsis), lung hypoplasia (congenital diaphragmatic hernia, oligohydramnios), and remodeled pulmonary vasculature [2]. Infants with PH and cardiac dysfunction are at an increased risk of decreased oxygenation in their body and their brain that might affect neurodevelopment. Numerous studies have shown that severe PH, myocardial dysfunction, and a continuous right-to-left shunt through the patent ductus arteriosus are associated with poor outcomes [5,6]. Importantly, various echocardiographic measures of the RV have been utilized for the early identification of risk and prognosis [7,8]. More recently, the right ventricular fractional area change (RV FAC), a surrogate marker for RV systolic function, has been shown to correlate with progression to death and the need for extracorporeal membrane oxygenation (ECMO) [9,10]. Impairments in motor development, cognition, and hearing have been identified in this population [4,11,12,13]; however, no studies have evaluated right ventricular echocardiographic metrics as a biomarker for neurodevelopment risk. The objective of this study was to determine if RV FAC can be an independent cardiac marker for the risk of short-term morbidity, mortality, and long-term neurodevelopmental impairment in infants with PH.

## 2. Materials and Methods

### 2.1. Design

This is a retrospective chart review of infants with PH who were admitted from January 2015 to December 2023 to the level IV neonatal intensive care unit (NICU) at the Children’s Hospital of Richmond (CHoR) at Virginia Commonwealth University (VCU) Health System. The echocardiographic report was reviewed to identify subjects with right ventricular dysfunction. The reports included subjective assessment by the reviewing cardiologist utilizing routine measurements—such as ventricular size, RV velocity, RV ejection fraction, etc.—to determine if subjects had RV dysfunction or not. Reports did not record tricuspid annular plane systolic excursion (TAPSE) or other measurements. This group was matched by gestational age and birth weight using the available convenience sample of subjects with PH whose echocardiographic report described right ventricular function as normal. The first objective of the study was to evaluate the long-term outcomes based on the subjective presence or absence of RV dysfunction. As part of this study, a cardiologist blinded to prior cardiologist echocardiogram reports reviewed the images to provide RV FAC values on all these subjects. The second objective of the study was to measure and compare the RV FAC in the groups with and without RV dysfunction. The third objective of the study was to evaluate if the RV FAC measurements, regardless of the function of RV, had any association with neurodevelopmental outcomes. Infants in both groups received standard treatment for PH targeted towards their clinical presentation and echocardiogram findings. This research study was approved by the VCU’s institutional review board (IRB).

### 2.2. Study Population

The study included infants born at ≥31 weeks’ gestation with an echocardiogram-confirmed diagnosis of pulmonary hypertension within the first 30 days of life. Infants transferred to the NICU after their seventh day of life or with any severe lethal malformation were excluded from the study.

### 2.3. Data Inclusion and Analysis

An extensive chart review of infants with a PH diagnosis was conducted. Maternal and neonatal demographic data including maternal age, prenatal care, race, ethnicity, gravidity, parity, tobacco and/or SSRI use during pregnancy, diabetes, hypertension, and asthma, as well as oligohydramnios, fetal pulmonary hypoplasia, delivery route, gestational age, birth weight, size for gestational age, infant’s sex, need for resuscitation at birth, and the Apgar score at 5 min of life were collected. Outcome measurements including incidence of neurodevelopmental impairment (primary outcome) as well as secondary outcomes such as the need for ventilatory support/duration of ventilatory support; use of inhaled nitric oxide (iNO); need for ECMO and its duration; need for sedation; acute/chronic kidney injury; use of pressors, sedation, and diuretics; as well as discharge outcomes including a need for tracheostomy, gastric tube (G tube)/nasogastric tube (NG tube) feeds at discharge, and the duration of hospitalization were collected.

Echocardiographic Evaluation: Pediatric cardiologist blinded to the patient’s record reviewed the cardiac images in infants with PH using a standardized imaging protocol. The RV end-diastolic area (RVEDA), the RV end-systolic area (RVESA) and other metrics were measured. The RV FAC, a bidimensional (longitudinal and transverse) measurement of the area change within the RV during each contraction, was calculated using the formula: RV FAC = (RVEDA − RVESA)/RVEDA. An RV FAC value of ≥0.35 (35%) is considered normal [7,9].

Neurodevelopmental Testing: Retrospective review included analysis of several standardized neurodevelopmental assessments based on postnatal age. The Test of Infant Motor Performance (TIMP) [14] was completed for infants < 17 weeks of adjusted age, and the Alberta Infant Motor Scale (AIMS) [15] was completed for infants between 18 weeks and 10 months of adjusted age. Results from the Bayley scales of infant and toddler development testing (third and fourth edition) [16], which were completed at one year of adjusted age and two years of chronological age, were also collected. Developmental domains measured by the Bayley included cognitive, language (receptive and expressive), and motor (fine and gross) skills. These neurodevelopmental outcome measures are administered as part of routine care in the NICU follow-up clinic. In our analysis, a value of one standard deviation below the mean was considered abnormal for all standardized tests.

### 2.4. Data Analysis

Comparisons of expected outcome levels between groups were carried out using the Wilcoxon rank sum test, Pearson’s chi-square test, and Fisher’s exact test. Multivariable linear regression analyses were performed to evaluate the correlation between the neurodevelopmental tests’ Z-scores and the RV FAC measurements. The established diagnoses reported in the literature that have been associated with NI, such as hypoxic-ischemic encephalopathy (HIE), GA < 35 weeks, and the presence of intraventricular hemorrhage (IVH), were controlled for using regression analyses. All continuous variables were tested for normality. Due to the exploratory nature of this study, a *p*-value < 0.05 was considered significant.

## 3. Results

### 3.1. Population Characteristics

**Figure 1:**
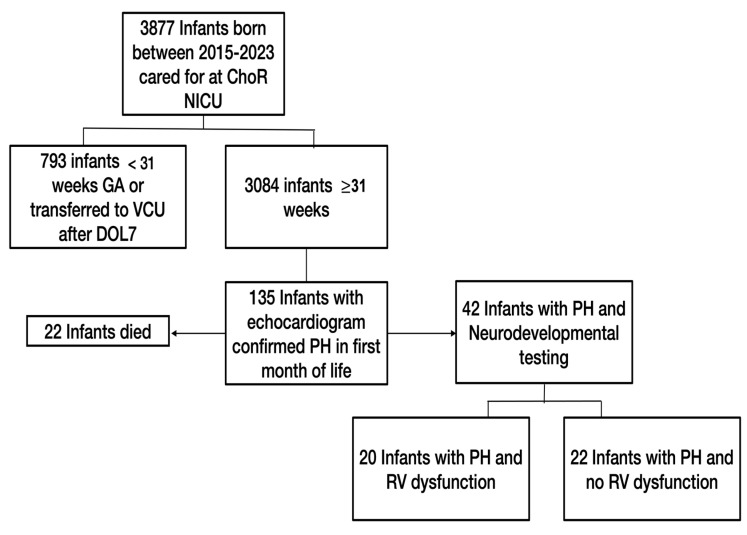
Flow diagram of study cohort.

There were 3877 infants admitted to the NICU from 2015 to 2023, and 3084 of those infants were born at ≥31 weeks’ gestation. Of these, 135 infants met the inclusion criteria of an echocardiogram-confirmed PH diagnosis within the first 30 days of life, with 42 infants having at least one formal neurodevelopmental outcome measured. Of the 42 identified infants, 20 infants (48%) had PH with RV systolic dysfunction, and 22 infants (52%) had PH with no RV systolic dysfunction (Figure 1).

Maternal demographics were similar among both groups (Table 1), with no significant difference in maternal comorbidities or known risk factors for the birth of an infant with PH. The average gestational age was 37 ± 2.6 weeks’ gestation, with a general male predominance (64%). There was, overall, higher cesarean delivery (55%), likely due to high-risk pregnancies (placental abruption, pre-eclampsia, meconium aspiration syndrome, etc.) causing fetal distress; however, no statistical difference was noted between the two groups. The average delivery Apgar score at 5 min was comparable in both groups (mean Apgar of 6.4), and the presence of neonatal comorbidities was not significantly different among the two groups (Table 2).

### 3.2. Assessment of the RV FAC as a Marker of RV Dysfunction

**Figure 2:**
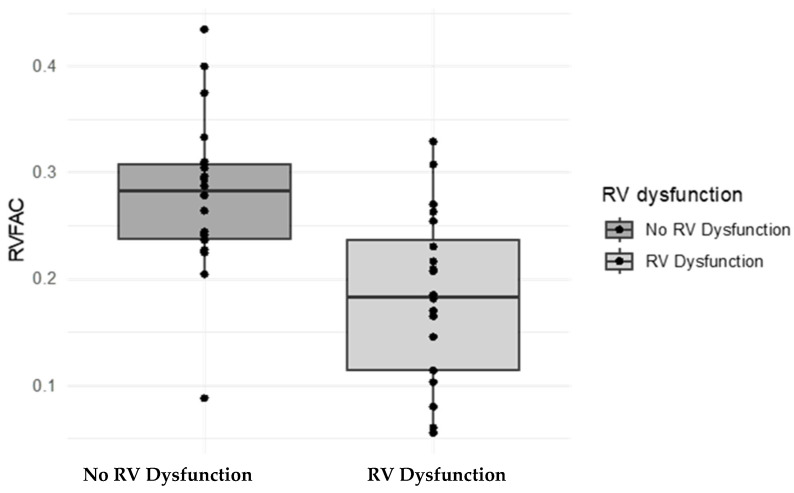
Correlation of the RV FAC with RV systolic dysfunction, N = 42. The horizontal axis represents the presence or absence of RV systolic dysfunction as determined by a subjective assessment based on additional measurements apart from the RV FAC; the vertical axis represents the RV FAC measurement (<0.35 is considered low in term infants).

Overall, RV FAC measurement was associated with the subjective assessment of ventricular systolic dysfunction provided by the pediatric cardiologist, demonstrating that it could potentially be considered a strong cardiac metric of RV dysfunction (Figure 2). In particular, the mean RV FAC was lower in children diagnosed with RV dysfunction. There was only one patient in the control group with normal RV function whose RV FAC was above 0.35. The mean RV FAC in the RV dysfunction group was 0.18 (0.20 ± 0.07) compared to 0.28 (0.23 ± 0.09) in infants with normal RV function and was statistically significant (*p* = 0.00017).

### 3.3. Neurodevelopmental Impairment in Cohort

**Figure 3:**
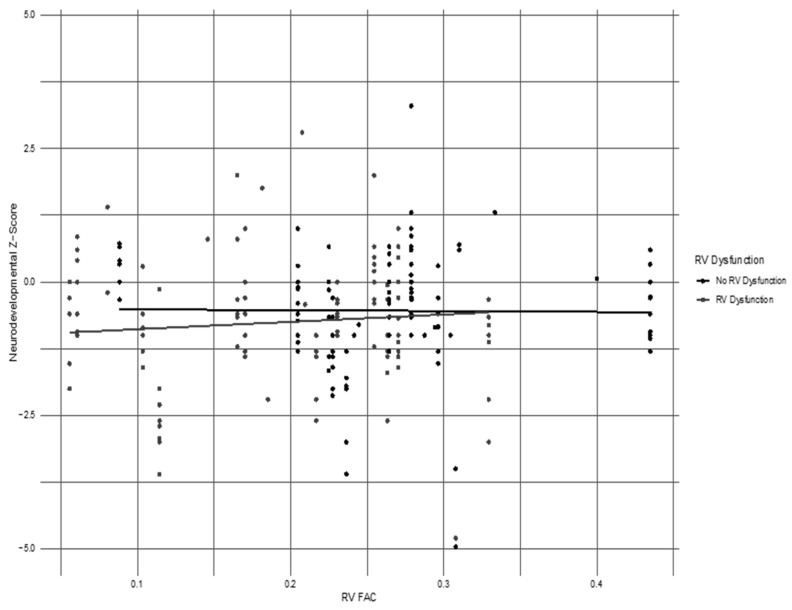
RV FAC vs neurodevelopmental outcome quantile-quantile plot. The horizontal axis represents the RV FAC measurement (<0.35 is considered abnormally low). The vertical axis represents ND test Z-scores.

Analyzing specific neurodevelopmental outcome measures, the overall percentage of subjects with neurodevelopmental impairment in any of the standardized assessments in the cohort at any point during their first two years of age was 62%. The NI in the PH with RV systolic dysfunction group was 60% (12/20), and 63% (14/22) in the group with no RV systolic dysfunction (*p*-value = 0.8). The details of the neurodevelopmental assessments performed at various ages, the identified delays, and the percentage of patients lost to follow-up are described in Table 3. We considered any abnormal value on a test to be a positive indication for neurodevelopmental delay. To account for the fact that not every test was taken by all subjects, we performed mixed effects regression analyses. There were also no statistical differences among infants with RV dysfunction or no RV dysfunction with regards to motor delay, language delay, or cognitive delay. The Z-scores of the regression analyses of standardized developmental testing for the TIMP, AIMS, and Bayley, controlled for a gestational age of <35 weeks, hypoxic-ischemic encephalopathy (HIE), and intraventricular hemorrhage, did not demonstrate statistical differences when compared with the RV FAC measurements in the combined cohort (Table 4). However, a lower RV FAC measurement was identified in those infants with a lower Z-score on their developmental outcome measure, though this did not reach statistical significance, as seen in the quantile-quantile plot in Figure 3.

### 3.4. Morbidities and Hospital Characteristics in Population Cohort

Mortality was noted to be 16.3% (22/135) in the initial study cohort of all subjects with PH. RV dysfunction was noted in 59% of the deceased infants. Surviving infants who were discharged from the NICU and had at least one standardized neurodevelopmental test performed were included in this study. Most infants in the study (69%) were depressed at birth, requiring some resuscitation intervention, but no statistical difference was noted between the groups with and without RV dysfunction when compared for the need for resuscitation, requirement, and duration of mechanical ventilation.

There was a longer need for sedation, higher need for pressors, higher need for ECMO, and longer length of hospital stay noted in the RV dysfunction group, though these did not reach statistical significance (Table 5). The use of milrinone was higher in infants with PH and RV systolic dysfunction (*p*-value < 0.002). The need for G tube feedings or NG feedings at discharge, as well as tracheostomy at discharge, were not significantly different among the groups (Table 5).

## 4. Discussion

Pulmonary hypertension is a systemic condition affecting multiple organs, making it a leading cause of morbidity and mortality in the neonatal population [17]. Cardiac dysfunction has been identified as a risk factor for worse clinical outcomes in pulmonary hypertension and for neurodevelopmental delays [6,18]. The echocardiographic evaluation of cardiac function varies from the subjective assessment by a cardiologist to well-defined, measurable indices. There are several cardiac metrics developed to estimate RV function—including tricuspid annular plane systolic excursion (TAPSE), RV strain, and RV ejection fraction (RVEF)—each with its own advantages and limitations, with no single marker prognosticating outcomes. TAPSE measures the longitudinal movement of the base of the heart toward the apex. TAPSE has limitations to its correlation with the RVEF, during an increase in RV afterload, as seen in pulmonary hypertension [7]. Recently, RV FAC has been advanced as a promising biomarker of RV dysfunction due to its ability to measure the transversal component of the RV contraction, which correlates better to the RVEF due to its measurement of septal bulging into the left ventricle [7]. In both preterm and healthy term infants, the RV FAC improves with postnatal age, with a value greater than 0.35 by one month of adjusted age [9,19]. For this study, a value > 0.35 is considered normal.

Infants with PH were divided into two cohorts: with or without subjective RV systolic dysfunction as described in the echocardiogram report during the clinical encounter. As part of this study, the RV FAC, which is a metric not routinely calculated during evaluation, was measured by a pediatric cardiologist blinded to the initial assessment. Even though an RV FAC of 0.35 is considered normal, in our cohort, all but one subject had a lower value. However, when both groups were compared, the mean RV FAC in the study group was statistically lower compared to the group with normal RV function. The lower RV FAC in our cohort could be due to known factors that could alter maturational changes in the RV, such as an early postnatal age and a lower weight at the time of assessment [19], or just the lack of a normal range of RV FACs in disease conditions like pulmonary hypertension and HIE. Giesinger et al. found TAPSE to be a better marker for NI in patients with HIE, though the sample size was small [18,20].

This study aimed to assess NI based on (i) the subjective assessment of RV function and (ii) the RV FAC, a valuable specific echocardiographic marker of RV dysfunction. We found no statistically significant difference in neurodevelopmental outcomes between groups based on RV dysfunction or lower RV FAC. However, we identified an overall higher incidence of NI (60%) in infants with PH compared to the 12% incidence reported by Rosenberg et al. [21], 25% reported by Konduri et al. [22] and 46% reported by Lipkin et al. [4]. The finding of a significantly higher incidence of motor, language, and cognitive delays can be explained by diagnostic and therapeutic differences in the identification and management of PH, the severity of PH in our sample, the neurodevelopmental assessment of infants who had a less severe clinical course and a potentially lower risk for NI, and an overall increase in survival.

The secondary outcome of this study was to evaluate the effect of RV dysfunction on short-term clinical outcomes in infants with pulmonary hypertension. There is limited information available in the literature to correlate RV dysfunction with clinical course. In our study, the use of milrinone was higher in infants with PH and RV systolic dysfunction (*p* < 0.05). Although not statistically significant, we found a need for longer sedation, higher pressors and ECMO, and longer hospital stays in the study’s RV dysfunction group. In infants with PH, regardless of RV dysfunction, the clinical outcome range reported by Jain [23] and Butt et al. [24] for the use of iNO (43–71%), inotropic support (80–88%), and the need for ECMO (10–27%) were comparable to our study. The requirements for oxygen at discharge (19%) and G tube feeds (14%) were similar to prior studies [11]. The mortality in our study was 16%, and a range of 4–33% has been reported in prior studies [2,8,17,25].

Our study had several limitations. It was a retrospective analysis with a small sample size of infants across different gestational ages and birth weights with echocardiograms performed at different postnatal ages within the first month of life. All these factors can affect the maturity of the right ventricle and, eventually, RV FAC measurement. RV dysfunction was based on subjective assessments and standardized markers, such as TAPSE etc., and were not measured or trended to evaluate RV function. The study sample included only those infants who had ND follow-ups, as PH is not a criterion for participation in a long-term follow-up clinic for neurodevelopmental assessment or for early intervention referral unless there is prolonged hospitalization. Most of the infants did not have neuroimaging, which could identify additional risks for NI. In our cohort, follow-up at 3–6 months of adjusted age was 92%, which decreased to 48% at 1 year and 19% by 2 years of age. There is a possibility that infants who were lost to follow-up could have had different outcomes at 2 years of age.

The findings in our study are relevant to other NICUs, as they re-confirm that pulmonary hypertension continues to be a high-risk diagnosis, needing both acute and long-term care. The significant finding of low RV FACs in infants with cardiac dysfunction suggests a need for additional studies seeking other cardiac markers to be used in conjunction with the RV FAC that could aid in determining clinical severity and prognosis. Even though the RV FAC cut-off value of 0.35 did not affect the neurodevelopmental outcomes in multiple regression analyses, a lower RV FAC may be more appropriate in newborns with PH. Further research is required to understand the role of cardiac dysfunction’s influence on neurodevelopmental risk to offer targeted preventive strategies to decrease long-term NI in this population. Importantly, infants with PH in the newborn period could be considered for postnatal neurodevelopmental evaluations for timely diagnosis and intervention of any impairment that could be remediated with early intervention services.

## 5. Conclusions

The results of these investigations demonstrate an overall developmental delay in 60% of infants with pulmonary hypertension, identifying significant neurodevelopmental risk in this population. The RV FAC was noted to be statistically lower in infants with RV dysfunction. Further studies are needed to validate the RV FAC as a surrogate marker for RV dysfunction. The occurrence of RV systolic dysfunction in infants with PH within the first 30 days of life was not an independent risk for developmental delay. Use of milrinone correlated with a lower RV FAC and impaired RV function and may be an indicator of RV dysfunction severity.

## Figures and Tables

**Table 1:** Maternal characteristics of the infant study cohort.

Groups				
Maternal Characteristics	Overall, *n* = 42 ^1^	No RV Dysfunction, *n* = 22 ^1^	RV Dysfunction, *n* = 20 ^1^	*p*-Value ^2^
Age	29.7 (5.4)	30.0 (4.8)	29.3 (6.1)	0.8
Race				0.2
White	21 (50%)	14 (64%)	7 (35%)	
Black	18 (43%)	7 (32%)	11 (55%)	
Other	3 (7.1%)	1 (4.5%)	2 (10%)	
Average BMI	34 (11)	32 (13)	37 (9)	0.10
Tobacco use during pregnancy	4 (9.5%)	3 (14%)	1 (5.0%)	0.6
SSRI use during pregnancy	4 (9.5%)	3 (14%)	1 (5.0%)	0.6
Asthma history	1 (2.4%)	0 (0%)	1 (5.0%)	0.5
Gestational hypertension	6 (14%)	2 (9.1%)	4 (20%)	0.4
Gestational diabetes	7 (17%)	4 (18%)	3 (15%)	>0.9
Prenatal steroids given	9 (21%)	7 (32%)	2 (10%)	0.13
Oligohydramnios	3 (7.1%)	1 (4.5%)	2 (10%)	0.6
Vaginal delivery	19 (45%)	9 (41%)	10 (50%)	0.6
C-section	23 (55%)	13 (59%)	10 (50%)	0.6

^1^ Based on available data; ^2^ Wilcoxon rank sum test, Pearson’s Chi-squared test, and Fisher’s exact test. All continuous variables were assessed for normality and reported as mean (sd).

**Table 2:** Infant characteristics of the study cohort.

Groups				
Infant Characteristics	Overall, *n* = 42 ^1^	No RV Dysfunction, *n* = 22 ^1^	RV Dysfunction, *n* = 20 ^1^	*p*-Value ^2^
Gestational age (weeks)	37.07 (2.64)	37.14 (2.66)	37.00 (2.70)	0.9
Gestational age less than or equal to 35 weeks	13 (31%)	7 (32%)	6 (30%)	0.9
Birth weight (grams)	2847 (780)	2665 (789)	3047 (737)	0.2
Size for gestational age				0.3
AGA	28 (67%)	15 (68%)	13 (65%)	
LGA	5 (12%)	1 (4.5%)	4 (20%)	
SGA	9 (21%)	6 (27%)	3 (15%)	
Male gender	27 (64%)	12 (55%)	15 (75%)	0.2
Apgar at 5 min (average score)	6 (2)	7 (3)	6 (2)	0.4
Apgar at 5 min (number with score less than 5)	8 (20%)	5 (23%)	3 (16%)	0.7
Intraventricular hemorrhage	9 (21%)	6 (27%)	3 (15%)	0.5
Seizures	8 (19%)	5 (23%)	3 (15%)	0.7
Respiratory distress syndrome	16 (38%)	10 (45%)	6 (30%)	0.3
Congenital heart disease	5 (12%)	1 (4.5%)	4 (20%)	0.2
Meconium aspiration syndrome	12 (29%)	7 (32%)	5 (25%)	0.6
Hypoxic-ischemic encephalopathy	10 (24%)	6 (27%)	4 (20%)	0.7
Pulmonary hypoplasia	4 (9.5%)	1 (4.5%)	3 (15%)	0.3

^1^ Based on available data; ^2^ Wilcoxon rank sum test, Pearson’s Chi-squared test, and Fisher’s exact test. All continuous variables were assessed for normality and reported as mean (sd).

**Table 3:** Neurodevelopmental impairment.

	RV Dysfunction			No RV Dysfunction		
	TIMP/AIMS 3–6 mo *n* = 18/20	Bayley 1 year *n* = 11/20	Bayley 2 years *n* = 4/20	TIMP/AIMS 3–6 mo *n* = 21/22	Bayley 1 year *n* = 9/22	Bayley 2 years *n* = 4/22
Gross motor delay % (n)	22 (4)	36 (4)	25 (1)	19 (4)	55 (5)	25 (1)
Fine motor delay % (n)	x	18 (2)	25 (1)	x	11 (1)	0
Expressive language delay % (n)	x	27 (3)	50 (2)	x	44 (4)	0
Receptive language delay % (n)	x	72 (8)	25 (1)	x	44 (4)	25 (1)
Cognitive delay % (n)	x	18 (2)	25 (1)	x	11 (1)	25 (1)
Lost to follow-up % (n)	10 (2)	45 (9)	80 (16)	0.5 (1)	59 (13)	81 (18)

**Table 4:** Multivariable linear regression analysis of the RV FAC and neurodevelopmental outcomes.

Neurodevelopment Test	RV FAC Coefficient	95% CI Lower	95% CI Upper	Standard Error	Neurodevelopment Z-Score	*p*-Value
TIMP	−0.50	−7.12	6.12	3.38	−0.15	0.88
AIMS	−39.73	−136.21	56.75	49.22	−0.81	0.45
Bayley cognitive	−0.50	−9.60	8.60	4.64	−0.11	0.91
Bayley receptive language	2.29	−2.31	6.89	2.35	0.98	0.34
Bayley expressive language	−0.24	−5.13	4.65	2.50	−0.10	0.92
Bayley comprehensive language	1.13	−3.88	6.14	2.56	0.44	0.66
Bayley fine motor	−1.02	−8.93	6.89	4.04	−0.25	0.80
Bayley gross motor	−5.48	−13.17	2.21	3.92	−1.40	0.18
Bayley comprehensive motor	−3.73	−12.33	4.87	4.39	−0.85	0.40

Note: Models controlled for the following variables: intraventricular hemorrhage, hypoxic-ischemic encephalopathy, and a gestational age of <35 weeks. TIMP—Test of Infant Motor Assessment; AIMS—Alberta Infant Motor Scale.

**Table 5:** Hospital interventions and short-term outcomes.

Groups				
Secondary Outcomes	Overall, *n* = 42 ^1^	No RV Dysfunction, *n* = 22 ^1^	RV Dysfunction, *n* = 20 ^1^	*p*-Value ^2^
Average pH level	7.22 (0.15)	7.24 (0.18)	7.20 (0.12)	0.2
Resuscitated at birth	29 (69%)	15 (68%)	14 (70%)	0.9
Therapeutic hypothermia performed	9 (21%)	5 (23%)	4 (20%)	>0.9
Inhaled nitric oxide	33 (79%)	17 (77%)	16 (80%)	>0.9
Duration of iNO (days)	5 (5)	5 (4)	6 (5)	0.3
ECMO performed	13 (32%)	5 (24%)	8 (40%)	0.3
Duration of ECMO (days)	3 (5)	2 (3)	4 (6)	0.2
Milrinone	17 (40%)	4 (18%)	13 (65%)	0.002
Vasopressors	20 (48%)	8 (36%)	12 (60%)	0.13
Diuretics	25 (60%)	11 (50%)	14 (70%)	0.2
Sedation	23 (55%)	11 (50%)	12 (60%)	0.5
Days of sedation	15 (23)	12 (16)	18 (29)	0.6
Mechanical ventilation required	38 (90%)	18 (82%)	20 (100%)	0.11
Days on mechanical ventilation	14 (14)	12 (12)	16 (16)	0.4
Oxygen required at discharge	8 (19%)	4 (18%)	4 (20%)	>0.9
Tracheostomy required	1 (2.4%)	1 (4.5%)	0 (0%)	>0.9
Gastrostomy tube required at discharge	6 (14%)	3 (14%)	3 (15%)	>0.9
Nasogastric tube required at discharge	8 (19%)	6 (27%)	2 (10%)	0.2
Length of stay (days)	50 (38)	45 (36)	56 (40)	0.3

^1^ Based on available data; ^2^ Wilcoxon rank sum test, Pearson’s Chi-squared test, and Fisher’s exact test. All continuous variables were assessed for normality and reported as mean (sd).

## Data Availability

Data is unavailable due to institutional privacy restrictions.
